# Psychometric properties of the perceived stress scale in a community sample of Chinese

**DOI:** 10.1186/s12888-020-02520-4

**Published:** 2020-03-20

**Authors:** Feifei Huang, Huijun Wang, Zhihong Wang, Jiguo Zhang, Wenwen Du, Chang Su, Xiaofang Jia, Yifei Ouyang, Yun Wang, Li Li, Hongru Jiang, Bing Zhang

**Affiliations:** National Institute for Nutrition and Health, Chinese Center for Disease Control and Prevention, Beijing, China

**Keywords:** Perceived stress, Reliability, Validity

## Abstract

**Background:**

The Perceived Stress Scale (PSS) is a globally used and self-report scale measuring perceived stress. Three versions of PSS (PSS-14, PSS-10 and PSS-4) are available which comprise 14, 10 and 4 items respectively. However, the Chinese version of the PSS has not yet been validated in a large community-based general population. The aims of this study were to evaluate the psychometric properties of the Chinese PSS in a large community-based general population and to compare the appropriateness of the three versions of PSS.

**Methods:**

A total of 9507 adults with at least a junior high school education and completed PSS-14 from the China Health and Nutrition Survey were involved in this study. The internal consistency reliability of PSS was assessed using Cronbach’s alpha coefficient and confirmatory factor analysis was employed to test the construct validity. Modification index was used for model extension and the critical ratio was used for model restriction.

**Results:**

The internal consistency coefficients were satisfactory for PSS-14 and PSS-10, but not for PSS-4. The corresponding Cronbach’s alpha were 0.830, 0.754 and 0.473 respectively. A 2-factor structure was confirmed for the PSS-14 and PSS-10, and all items’ standardized factor loadings exceeded 0.4 for either negative or positive factors. Given that item 12 loaded on both negative and positive factors for PSS-14 and the goodness of fit for PSS-14 was not acceptable, PSS-13 (PSS-14 excluding item 12) was studied. The construct validities of PSS-13 and PSS-10 were satisfactory, but the goodness of fit for PSS-10 were better than that for PSS-13.

**Conclusions:**

PSS-13 (PSS-14 excluding item 12) and PSS-10 have satisfactory psychometric properties. PSS-10 are more applicable to measure the perceived stress than PSS-13 in a large community-based general population in China.

## Background

The concept of stress can be classified approximately into three perspectives which are environmental, psychological and biological stress [1]. Previous studies have shown that psychological stress is associated with eating behavior, smoking, physical activity, waist circumference, BMI and other health outcomes [2–6]. Perceived Stress Scale (PSS), developed by Cohen, Kamarck and Mermelstein [7], is one of the most widely used tools to measure psychological stress in the world. Instead of focusing on a particular event, the PSS appraises the extent that the participants feel unpredictable, uncontrollable or overloaded in their lives [8]. The original PSS comprises 14 items (PSS-14). Two shorten versions (PSS-10 and PSS-4) are also available which comprise 10 and 4 items selected from the PSS-14 respectively [7, 8].

The PSS has been translated into many languages and the reliability and validity have been verified in different countries. For instance, PSS-14 has been evaluated in Sweden [9], France [10], Greece [11], Mexico [12], America [13] and Japan [14]; PSS-10 has been evaluated in Sweden [15], France [10], Korea [16], Mexico [12], America [13], Arabia [17], Serbia [18], Germany [19], Vietnam [20], Brazil [21] and Thailand [22]; PSS-4 has been evaluated in France [10], Korea [16], Mexico [12] and America [13]. In China, a few studies have verified the PSS in specific population, such as policewomen (PSS-10) [23], university students (PSS-10) [24], elderly service workers (PSS-10) [25] and cardiac patients who smoke (PSS-14, 10 and 4) [26]. It is limited when generalize the PSS for use with other population. Therefore, the objective of this study is to evaluate the psychometric properties of the PSS in a large general community-based population in China and to evaluate the appropriateness of the three versions of PSS.

## Methods

### Participants

The participants were from the China Health and Nutrition Survey co-operated by the National Institute for Nutrition and Health of the Chinese Center for Disease Control and Prevention and the University of North Carolina at Chapel Hill in the United States [27]. The ongoing open cohort began in 1989 and drew a sample using a multistage, random cluster sampling method. There were eight diverse provinces and autonomous regions from 1989 to 1997, nine from 2000 to 2009, three municipalities were added in 2011, three provinces were added in 2015. The 2015 survey is the first survey to incorporate Perceived Stress Scale (PSS). The most recent database in 2018 has not been released, hence the present study uses data from the 2015 wave.

The PSS was designed for use with community samples with at least a junior high school education [7]. Therefore, we included participants with age ≥ 18 years and education ≥ junior high school, and excluded those who did not complete the PSS. There were 10,798 individuals with age ≥ 18 years and education ≥ junior high school. 1291 individuals with uncompleted PSS were excluded. There were 9507 individuals at 5296 households living in 361 communities involved in this study eventually. The institutional review board of the University of North Carolina at Chapel Hill and the National Institute for Nutrition and Health, Chinese Center for Disease Control and Prevention approved the study protocol (ethics approval code 201524). All of the participants signed the informed consents.

### Measures

The original PSS consists of 14 items (PSS-14) which was translated from English into Chinese, and subsequently back into English to ensure the accuracy of translation. Each item is rated on a 5-point Likert-type scale, ranging from 0 = ‘never’ to 4 = ‘very often’. The scale can cluster into two subscales: negative subscale (items 1,2,3,8,11,12 and 14) and positive subscale (items 4,5,6,7,9,10 and 13)**.** The negative subscale negatively states items(e.g., In the last month, how often have you been upset because of something that happened unexpectedly?), and is intended to assess lack of control and negative reactions (perceived distress), while the positive subscale positively states items(e.g., In the last month, how often have you dealt successfully with irritating life hassles?), and measures the degree of ability to cope with existing stressors (coping capacity) [7, 9, 26]. PSS-10, a shorter version of PSS-14, comprises six negative (items 1,2,3,8,11 and 14) and four positive items (items 6,7,9 and 10). PSS-4, designed for telephone interviews, has four items (items 2,6,7 and 14). The total score of PSS is obtained by reversing the scores on the positive items and then summing across all the items, with a higher score indicating higher perceived stress. Possible total scores for PSS-14, PSS-10 and PSS-4 range from 0 to 56, 0 to 40 and 0 to 16 respectively. In our study, we asked the participants to answer the PSS-14, and then calculate the total scores of PSS-14, PSS-10 and PSS-4 respectively according to the corresponding items.

### Statistical analysis

The internal consistency reliability of the three versions of PSS was examined by Cronbach’s alpha and the reasonable acceptability criterion of which is ≥0.70.

The construct validity was examined by confirmatory factor analysis (CFA). Using the generalized least squares method, the two-factor models were fitted for different versions of PSS respectively to assess the goodness-of-fit of the factor structure. Models with goodness-of-fit index (GFI) > 0.9, adjust goodness-of-fit index (AGFI) > 0.9, comparative fit index (CFI) > 0.9, standardized root mean square residual (SRMR) < 0.08, and root mean square error of approximation (RMSEA) < 0.08 were regarded as a good fit. Little suggested that investigators ought not rely too heavily on chi-square test for comparing competing models, but rather on the indices mentioned above to determine the overall adequacy of a fitted model, for the chi-square value was an overly sensitive index of fit when working with large measurement models. Therefore, we reported chi-square value, freedom degree and corresponding *P* value to ensure the results’ completeness.

The CFA analyses were performed by Amos 24.0. Cronbach’s alpha was obtained using SPSS 21.0. All statistical tests were two-tailed and employed a significance level at *p* < 0.05.

### Model modifications

Two kinds of model modification indices were used, of which the modification index(M.I.) was used for model extension and the critical ratio(C.R.) was used for model restriction. Modification priority was given to the path with the maximum M.I. value or C.R. value.

## Results

### The sample demographics

The sample consisted of 9507 individuals with a mean age of 47.5 years (SD = 14.1) and 51.1% of the sample were men. The majority (88.4%) of the participants were married. The demographics are presented in Table 1. The mean scores of the PSS-14, PSS-10 and PSS-4 reported in this sample were 27.5 ± 7.1, 19.2 ± 4.9 and 8.0 ± 2.2 respectively.

**Table 1:** Sample demographics

	Total sample	Men	Women
Age (y, mean ± SD)	47.5 ± 14.1	49.0 ± 14.3	46.0 ± 13.7
Net individual income (median, yuan)	24,000.0	24,000.0	24,000.0
Education (n,%)			
Middle school	4450 (46.8)	2242 (46.1)	2208 (47.5)
High school	3065 (32.2)	1599 (32.9)	1466 (31.6)
College and above	1992 (21.0)	1020 (21.0)	972 (20.9)
Marital status (n,%)			
Unmarried	641 (6.8)	366 (7.6)	275 (6.0)
Married	8300 (88.4)	4277 (89.2)	4023 (87.6)
Others	566 (4.8)	218 (3.2)	348 (6.4)
Region (n,%)			
Urban	4174 (32.9)	2012 (41.4)	2162 (46.5)
Rural	5333 (56.1)	2849 (58.6)	2484 (53.5)
Work (n,%)			
Employed	5254 (55.3)	2994 (61.6)	2260 (48.6)
Seeking work	348 (3.7)	248 (5.1)	100 (2.2)
Doing housework	1492 (15.7)	372 (7.7)	1120 (24.1)
Retired	1684 (17.7)	788 (16.2)	896 (19.3)
Others	729 (7.7)	459 (9.4)	270 (5.8)
Sample size (n,%)	9507 (100.0)	4861 (100.0)	4646 (100.0)

### Internal consistency reliability

The Cronbach’s alpha was 0.830 (0.813 and 0.882 for the negative and positive subscales, respectively) for the PSS-14 and 0.754 (0.820 and 0.865 for the negative and positive subscales, respectively) for the PSS-10. When each item of the PSS − 14 and PSS-10 was deleted from the analysis in order to test the robustness, Cronbach’s alpha remained high (0.811–0.829 for the PSS-14 and 0.728–0.739 for the PSS-10). The Cronbach’s alpha was 0.473 for the PSS-4. When each item of the PSS − 4 was excluded, Cronbach’s alpha ranged from 0.295 to 0.495.

### Confirmatory factor analysis

**Fig. 1:**
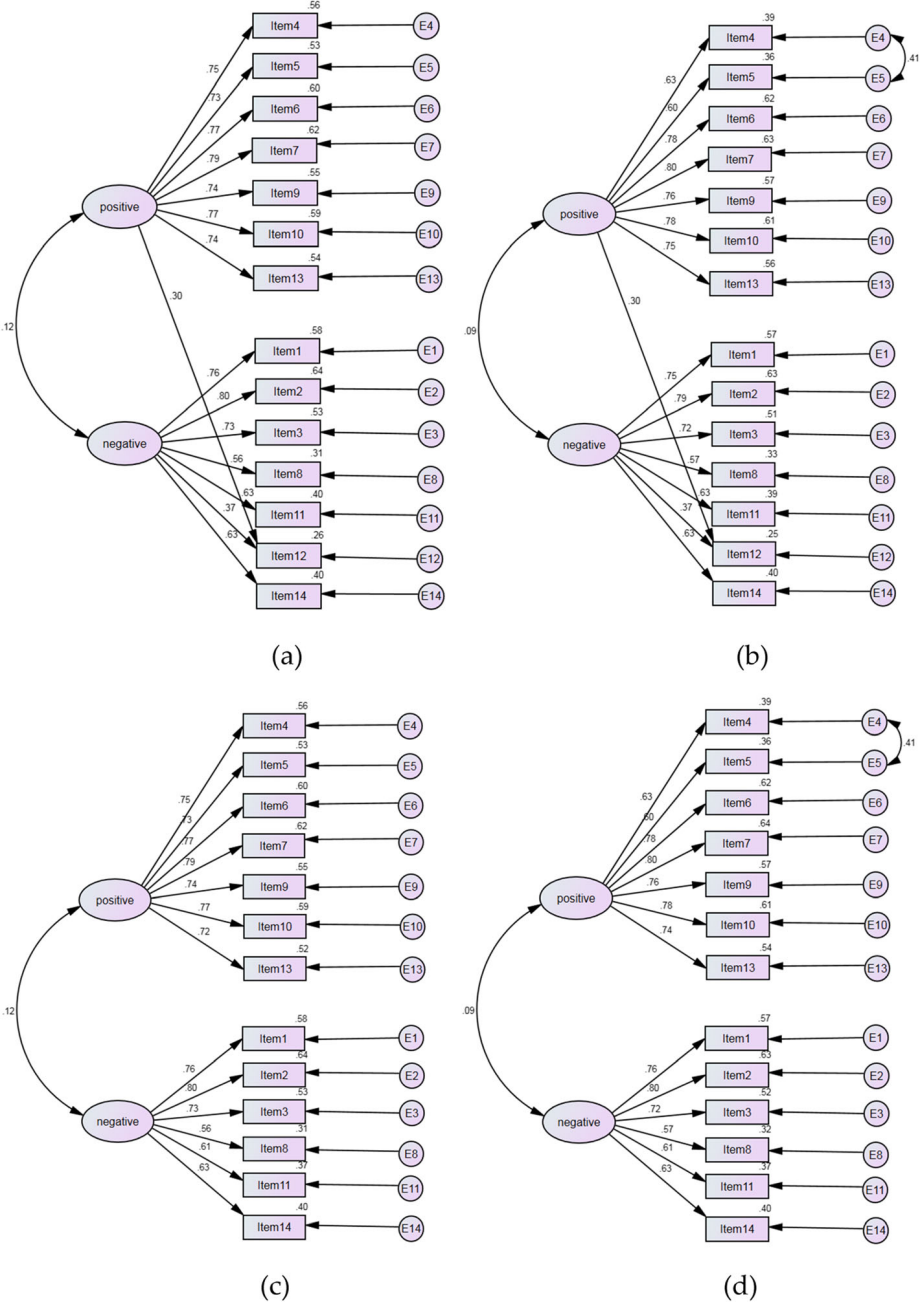
**a** Models of modified PSS-14-a; **b** Models of modified PSS-14-b; **c** Models of PSS-13; **d** Models of modified-PSS-13

The goodness-of-fit indices of confirmatory factor analysis (Table 2) presented that the 2-factor model did not fit well with PSS-14 (GFI = 0.923, AGFI = 0.894, CFI = 0.548, RMR = 0.107, SRMR = 0.092 and RMSEA = 0.083). After adding the path from positive factor to item 12 in the model (see modified PSS-14-a), the fitness was acceptable and AIC decreased from 5155.516 to 4503.156. After adding the two-way path between error 4 and error 5 based on the modified PSS-14-a (see modified PSS-14-b), all of the goodness-of-fit indices improved (GFI = 0.947, AGFI = 0.925, CFI = 0.697, RMR = 0.060, SRMR = 0.064 and RMSEA = 0.070) and AIC decreased from 4503.156 to 3579.504 again. The 2-factor model fitted marginally with PSS-13 in which the item 12 was deleted. After adding the two-way path between error 4 and error 5 in the model (see modified PSS-13), the fitness greatly improved. As for PSS-10, the 2-factor model was satisfactory (GFI = 0.959, AGFI = 0.936, CFI = 0.778, RMR = 0.054, SRMR = 0.055 and RMSEA = 0.076) and did not need to be modified. Although in all models, the ratio of chi-square value to degrees of freedom was beyond the range of 1–3, it did not matter heavily in such a large sample as this study. Figure 1 visualized the models in order to clearly understand their structure.

**Table 2:** Goodness-of-fit indices of confirmatory factor analyses

Models	χ^2^	df	*p*-value	CMIN/DF	GFI	AGFI	CFI	RMR	SRMR	RMSEA	AIC
PSS-14	5097.516	76	< 0.001	67.073	0.923	0.894	0.558	0.107	0.092	0.083	5155.516
Modified PSS-14-a	4443.156	75	< 0.001	59.242	0.933	0.907	0.616	0.069	0.074	0.078	4503.156
Modified PSS-14-b	3517.504	74	< 0.001	47.534	0.947	0.925	0.697	0.060	0.064	0.070	3579.504
PSS-13	4088.353	64	< 0.001	63.881	0.934	0.906	0.633	0.072	0.077	0.081	4142.353
Modified PSS-13	3164.756	63	< 0.001	50.234	0.949	0.926	0.717	0.061	0.065	0.072	3220.756
PSS-10	1942.359	35	< 0.001	55.496	0.959	0.936	0.778	0.054	0.055	0.076	1982.359

*df* degrees of freedom, *DMIN/DF* ratio of chi-square value to degrees of freedom, *GFI* goodness-of-fit index, *AGFI* adjusted goodness-of-fit index, *CFI* comparative fit index, *SRMR* standardized root mean square residual, *RMSEA* root mean square error of approximation, *AIC* akaike information criterion, *PSS-14* perceived stress scale with 14 items; Modified PSS-14-a: path from positive factor to item 12 was added to PSS-14; Modified PSS-14-b: two-way path between error 4 and error 5 were added to modified PSS-14-a; PSS-13: item 12 was deleted from PSS-14; Modified PSS-13: two-way path between error 4 and error 5 was added to PSS-13; PSS-10: perceived stress scale with 10 items.

Table 3 revealed that all of the standardized factor loadings were statistically significant in PSS-14, modified PSS-14-a, PSS-13 and PSS-10. The standardized factor loadings ranged from 0.471 to 0.798 for negative factor and from 0.663 to 0.759 for positive factor in PSS-14 and the between-factors correlation was 0.200. Item 12 loaded highly on both factors (0.375 for negative and 0.297 for positive factor respectively). After deleting item 12, we can see that all of the remaining loadings were > 0.5 in PSS-13. For PSS-10, all of the standardized factor loadings were > 0.6 for negative factor except item 8 (0.495) and > 0.7 for positive factor. The between-factors correlation was not significant in PSS-10 (*P* = 0.819).

**Table 3:** Standardized factor loadings of the 2-factor models

Item	PSS-14		Modified PSS-14-a		PSS-13		PSS-10	
	Negative factor	Positive factor	Negative factor	Positive factor	Negative factor	Positive factor	Negative factor	Positive factor
1	0.760^*^		0.761^*^		0.765^*^		0.781^*^	
2	0.798^*^		0.800^*^		0.802^*^		0.823^*^	
3	0.729^*^		0.728^*^		0.729^*^		0.749^*^	
8	0.565^*^		0.561^*^		0.559^*^		0.495^*^	
11	0.628^*^		0.629^*^		0.611^*^		0.607^*^	
12	0.471^*^		0.375^*^	0.297^*^	–		–	–
14	0.633^*^		0.634^*^		0.630^*^		0.633^*^	
4		0.740^*^		0.751^*^		0.752^*^	–	–
5		0.710^*^		0.727^*^		0.726^*^	–	–
6		0.759^*^		0.775^*^		0.775^*^		0.787^*^
7		0.759^*^		0.787^*^		0.787^*^		0.804^*^
9		0.706^*^		0.745^*^		0.745^*^		0.756^*^
10		0.721^*^		0.770^*^		0.771^*^		0.768^*^
13		0.663^*^		0.736^*^		0.724^*^	–	–
Factor correlation	0.200^*^		0.123^*^		0.120^*^		−0.003(P = 0.819)	

^*^: *P* < 0.01; PSS-14: perceived stress scale with 14 items; Modified PSS-14-a: path from positive factor to item 12 was added; PSS-13: item 12 was deleted from PSS-14; PSS-10: perceived stress scale with 10 items

### Comparison of stress level by characteristics

Table 4 described the stress level as measured by PSS-10 and the statistical test results by age, gender and work. Mean scores on the PSS-10 for men, women and the total samples (men and women combined) were 19.1, 19.3 and 19.2 respectively. Standard deviations were 5.0, 4.8 and 4.9 respectively. The mean scores for men and women didn’t significantly differ (*P* = 0.169). The mean score significantly decreased with age from 19.6 in the 18–44 age group to 18.6 in the 60–94 age group (*P* < 0.001). In addition, the PSS-10 score of participants who were employed was the highest.

**Table 4:** Means of total scores on PSS-10 by age, gender and work

	Sample size		Total		*P*
	n	%	Mean	SD	
Age					
18–44	4161	43.8	19.6	4.7	< 0.001^*^
45–59	3426	36.0	19.2	5.0	
60–94	1920	20.2	18.6	5.1	
Gender					
Men	4861	51.1	19.1	5.0	0.169^**^
Women	4646	48.9	19.3	4.8	
Work					
Employed	5254	55.3	19.5^a^	4.6	< 0.001^*^
Seeking work	348	3.7	18.7^bc^	5.6	
Doing housework	1492	15.7	19.2^ab^	5.1	
Retired	1684	17.7	18.6^c^	5.1	
Total sample	9507	100.0	19.2	4.9	–

^*^Kruskal-Walls rank sum test used for comparing mean differences in the total score by age

^**^Wilcoxon rank sum test used for comparing mean differences in the total score by gender

^a,b,c^results of LSD test; different letters indicate significant differences between groups

## Discussion

This study verified the reliability and construct validity of the Chinese version of the Perceived Stress Scale (PSS-14, PSS-10 and PSS-4). To our knowledge, this study is the first to evaluate the psychometric properties of the PSS in a large general community-based population in China. The results presented that the PSS-10 and modified PSS-14 were suitable for this population, while PSS-14 and PSS-4 did not have adequate psychometric properties.

Cronbach’s alpha values in this current study revealed that not only the overall PSS-14 and PSS-10, but also each of the two subscales of PSS-14 and PSS-10 were internally reliable, but PSS-4 was not. These findings were in line with previous studies in different countries, such as China [23], Japan [14], Vietnam [20], Korea [28], Thailand [22], Arabia [17], America [29, 30], Brazil [21], Greece [11, 31], Mexico [12], Germany [19], Sweden [9, 15] and Serbia [18]. Few study showed acceptable reliability of PSS-4, such as the United Kingdom [32], French workers [10] and American survivors of suicide [13]. Two studies found that the Cronbach’s alpha did not meet the Kline’s criteria, but the authors believed that the PSS-4 was reliable for some other reasons, for example the item-total correlations and split-half coefficient were high [16], and a reliability coefficient as low as 0.5 should not seriously attenuate validity [26]. We thought that PSS-4 when applied in our population was not reliable, hence we did not analyze its validity.

Our study supported a two-factor structure of the 14-, 13- and 10-item versions of PSS which was confirmed by most previous studies [18–20, 23, 26, 28, 29]. As expected, the two factors in our study also represented negative and positive feelings, because all the negatively worded items loaded together and all the positively worded items loaded together. In line with some studies [9, 26, 29, 31], our results showed that item 12 (In the last month, how often have you found yourself thinking about things that you have to accomplish?) had relatively low factor loading and loaded approximately equally on both negative and positive factors. This might be due to the translation or the potential interpretation by the subjects, but the possibility needed to be verified in further studies utilizing the Chinese versions of PSS. Given the item 12 was not a good measure for either of the subscale for PSS-14, some researchers suggested delete this item when calculating the total score or subscale scores in future studies [9, 29]. We compared the modified PSS-14-a, which had one more path from positive factor to item 12 than PSS-14 to PSS-13, and found that the fitness of PSS-13 was better. Therefore, we also proposed that item 12 be deleted. With regard to the PSS-10, all of items highly loaded on their designated factors. Although most of the previous studies confirmed the two-factor model of PSS, it was controversial whether using the full scale as a whole or using the two sub-scales separately. Considering the correlation between the two factors, some researchers recommended using the scale as a whole [8, 21, 23], while others suggested using the two factors as separate indicators of stress although which were weakly correlated [29]. In our study, the two factors were weakly correlated for PSS-13(r_s_ = 0.120), and were not correlated for PSS-10, we believed that it was acceptable no matter using the sub-scales as a whole or using them separately.

By confirmatory factor analysis, our study found covariance between error terms of items 4 and 5 indicating a systematic error in the response. The existence of error covariance may be due to the high degree of overlap in item content, but it was unclear. However, it was seemingly unlikely due to the subjects’ misunderstanding of item 4 (“In the last month, how often have you dealt successfully with irritating life hassles?”) and item 5(“In the last month, how often have you felt that you were effectively coping with important changes that were occurring in your life?”), because “irritating life hassles” was totally different from “important changes”. More studies were need to analyze the error covariance.

Although the psychometric properties of both PSS-13 and PSS-10 were satisfactory, the reliability and validity of PSS-10 were the best when compared to PSS-13. Moreover, it was critical to complete the questionnaire in a shorter time in a large survey with abundant multiple measures. Therefore, we recommended measurement of perceived stress utilizing the PSS-10 among the community-based general population in China.

The first advantage of this study is our utilization of a large community-based general Chinese population. The second advantage is that we excluded those with education lower than junior high school, to whom the PSS is not applicable. To our knowledge, no authors have mentioned this in their manuscripts. There are a few limitations. First, the China Nutrition Transition Cohort Study does not involve other psychological investigation. We can only verify the structure validity of PSS, but cannot verify the concurrent validity or other validity.

## Conclusions

Comprehensively, the results of our study reveal that PSS-13 (PSS-14 excluding item 12) and PSS-10 have satisfactory psychometric properties. PSS-10 are more applicable to measure the perceived stress than PSS-13 in a large community-based general population in China.

## Data Availability

The datasets generated and analyzed during the current study are available in the Carolina Population Center repository, http://www.cpc.unc.edu/projects/china/data.

## Abbreviations

AGFI: Adjust goodness-of-fit index
C.R.: Critical ratio
CFA: Confirmatory factor analysis
CFI: Comparative fit index
GFI: Goodness-of-fit index
M.I.: Modification index
PSS: Perceived stress scale
RMSEA: Root mean square error of approximation
SRMR: Standardized root mean square residual
