# Correction to: Psychometric properties of the perceived stress scale in a community sample of Chinese

**DOI:** 10.1186/s12888-020-02670-5

**Published:** 2020-05-25

**Authors:** Feifei Huang, Huijun Wang, Zhihong Wang, Jiguo Zhang, Wenwen Du, Chang Su, Xiaofang Jia, Yifei Ouyang, Yun Wang, Li Li, Hongru Jiang, Bing Zhang

**Affiliations:** National Institute for Nutrition and Health, Chinese Center for Disease Control and Prevention, Beijing, China

**Correction to: BMC Psychiatry 20, 130 (2020)**

**https://doi.org10.1186/s12888-020-02520-4**

Following publication of the original article [1], the authors noticed that the description of stress level and the Cronbach’s alpha values were incorrect. The corrections are listed below and they do not affect the conclusions. The authors regret any inconvenience that these inaccuracies might have caused.

1. Page 1: The Cronbach’s alpha of PSS-14, PSS-10 and PSS-4 were 0.738, 0.728 and 0.518 respectively in the Results section of the abstract.

2. Page 3: In the sample demographics section of results, the mean scores of the PSS-14, PSS-10 and PSS-4 reported in this sample were 22.7 ± 6.1, 15.4 ± 4.7 and 5.9 ± 2.3.

3. Page 3: The internal consistency reliability section of results should read as follows, ‘The Cronbach’s alpha was 0.738 (0.813 and 0.882 for the negative and positive subscales, respectively) for the PSS-14 and 0.728 (0.820 and 0.865 for the negative and positive subscales, respectively) for the PSS-10. When each item of the PSS-14 and PSS-10 was deleted from the analysis in order to test the robustness, Cronbach’s alpha remained high (0.698-0.765 for the PSS-14 and 0.687-0.736 for the PSS-10). The Cronbach’s alpha was 0.518 for the PSS-4. When each item of the PSS-4 was excluded, Cronbach’s alpha ranged from 0.347 to 0.536’.

4. Page 4: The comparison of stress level by characteristics section should read as follows, ‘Table 4 described the stress level as measured by PSS-10 and the statistical test results by age, gender and work. Mean scores on the PSS-10 for men, women and the total samples (men and women combined) were 15.4, 15.4 and 15.4 respectively. Standard deviations were 4.8, 4.7 and 4.7 respectively. The mean scores for men and women didn’t significantly differ (*P* = 0.559). The mean score significantly decreased with age from 15.7 in the 18–44 age group to 14.9 in the 60–94 age group (*P* < 0.001). In addition, the PSS-10 score of participants who were seeking work was the highest’.

**Table 4:** Means of total scores on PSS-10 by age, gender and work

	Sample size		Total		*P*
	n	%	Mean	SD	
Age					
18–44	4161	43.8	15.7	4.5	< 0.001^*^
45–59	3426	36.0	15.4	4.8	
60–94	1920	20.2	14.9	4.9	
Gender					
Men	4861	51.1	15.4	4.8	0.559^**^
Women	4646	48.9	15.4	4.7	
Work					
Employed	5254	55.3	15.1 ^a^	4.7	< 0.001^*^
Seeking work	348	3.7	17.0 ^a^	3.9	
Doing housework	1492	15.7	16.6 ^b^	4.2	
Retired	1684	17.7	14.5 ^c^	5.0	
Total sample	9507	100.0	15.4	4.7	–

* Kruskal-Walls rank sum test used for comparing mean differences in the total score by age

** Wilcoxon rank sum test used for comparing mean differences in the total score by gender

a,b,c results of LSD test; different letters indicate significant differences between groups

5. Page 6: In Table 3, the factor correlation of negative factor and positive factor for PSS-10 should be 0.003.

6. Page 6: The Table 4 should be replaced as follows.
